# Exploring Ethnic Variability in Aryl Hydrocarbon Receptor Signaling: Delineating Differences in Prostate Cancer Outcomes Between African American and Caucasian Populations

**DOI:** 10.7759/cureus.72474

**Published:** 2024-10-27

**Authors:** Gurjot Singh, Shubam Trehan, Anupam Singh, Kanwarpreet S Sandhu, Pratiksha Ratnani, Prateek Jain, Tejal Mehta, FNU Kalpana, Amna Farooq, Shipra Sharma, Gaurav Bector, Aayush Jain

**Affiliations:** 1 Internal Medicine, Maharaj Sawan Singh (MSS) Charitable Hospital, Beas, IND; 2 Internal Medicine, Dayanand Medical College and Hospital, Ludhiana, IND; 3 Internal Medicine, Yale New Haven Hospital, New Haven, USA

**Keywords:** african american, androgen receptor, androgen receptor signaling, aryl hydrocarbon receptors, caucasian, ethnic disparities, genetics, genomic studies, prostrate cancer

## Abstract

Prostate cancer rates and outcomes show significant differences between African American (AA) and Caucasian men, with AA males experiencing higher incidence and mortality rates. These disparities result from a complex interaction of socioeconomic, environmental, and biological factors. This study explores how the Aryl Hydrocarbon Receptor (AHR) and Androgen Receptor (AR) signaling pathways contribute to these differences.

AHR, traditionally recognized for its role in detoxifying environmental carcinogens, has recently been identified as playing a key role in prostate cancer progression. AA men tend to exhibit higher levels of AHR expression and activity, which may contribute to the aggressive nature of the disease in this population. The interaction between AHR and AR signaling pathways might promote tumor growth and lead to resistance to standard treatments. Additionally, genetic variations in the AHR and AR genes, along with environmental exposures, may exacerbate these disparities. This study emphasizes the importance of developing targeted therapies that address the specific genetic and molecular profiles of different populations. By gaining a deeper understanding of the roles of AHR and AR signaling in prostate cancer, particularly in the context of ethnic diversity, we aim to work toward reducing these disparities and improving outcomes for all patients.

## Introduction and background

Prostate cancer is the most frequently diagnosed non-skin cancer in men worldwide and represents a significant public health challenge, particularly in the United States, where it is the second leading cause of cancer-related deaths among men [1]. However, the distribution of prostate cancer incidence is not uniform across different populations, with African American (AA) men experiencing a disproportionately high burden of the disease. AA men have a 1.6 times higher risk of being diagnosed with prostate cancer and are 2.4 times more likely to die from the disease compared to their Caucasian counterparts [2-3]. These disparities are driven by a complex interplay of socioeconomic, environmental, and biological factors. Despite extensive research, the exact factors contributing to these differences remain unclear, necessitating deeper exploration into the biological mechanisms that may underlie these disparities.

Research increasingly points to intrinsic biological factors, including genetic and molecular differences, as significant contributors to the disparities observed in prostate cancer outcomes. Among these, the Aryl Hydrocarbon Receptor (AHR), a ligand-activated transcription factor initially associated with detoxifying environmental pollutants, has emerged as a critical player in prostate cancer biology [4]. AHR is involved in various cellular processes relevant to cancer, including cell cycle regulation, modulation of the immune response, and interaction with hormone receptor signaling pathways, particularly the Androgen Receptor (AR) signaling pathway, which is central to prostate cancer progression [5-6]. This interaction between AHR and AR signaling is especially important in the context of ethnic disparities, as AA men have been shown to exhibit higher levels of AR and possess unique genetic polymorphisms associated with AR, which may contribute to the more aggressive prostate cancer phenotype observed in this population [7-8].

Traditionally, AHR’s role has been understood in the context of mediating the effects of environmental carcinogens, such as polycyclic aromatic hydrocarbons and dioxins [9]. Upon ligand binding, AHR translocates to the nucleus, where it dimerizes with the AHR Nuclear Translocator (ARNT) and binds to specific DNA sequences known as xenobiotic response elements (XREs), regulating the expression of target genes. These genes include cytochrome P450 enzymes involved in xenobiotic metabolism, and genes associated with cell proliferation, differentiation, and apoptosis [10]. However, recent research has expanded the significance of AHR beyond its role in xenobiotic metabolism, suggesting its involvement in tumorigenesis in various cancers, including prostate cancer [11-12].

Importantly, AHR signaling has been shown to influence several aspects of prostate cancer progression, including AR signaling, cell proliferation, and invasiveness [13]. AHR expression is upregulated in androgen-independent prostate cancer cells, indicating its role in maintaining hormone-independent proliferation, a characteristic associated with more aggressive and treatment-resistant forms of prostate cancer [14]. Furthermore, AHR has been linked to the regulation of inflammatory pathways and immune responses within the tumor microenvironment, both of which are known to significantly influence cancer progression and may vary across different ethnic groups [15-16].

Differences in AHR signaling pathways may contribute to the observed ethnic disparities in prostate cancer outcomes. Recent studies suggest that AA men may exhibit higher levels of AHR expression in prostate cancer tissues, potentially contributing to more aggressive tumor behavior [17]. Genetic studies have identified certain AHR variants that are more prevalent in AA populations, suggesting a potential genetic basis for the differences in AHR activity and its impact on prostate cancer development [18]. These findings underscore the need for a more nuanced understanding of AHR’s role in prostate cancer, particularly in the context of ethnic diversity.

Beyond its interaction with AR signaling, AHR has also been shown to engage with other cancer-relevant signaling pathways, including the Wnt/β-catenin, NF-κB, and MAPK pathways [19]. These pathways regulate key processes such as cell proliferation, survival, and migration, and their dysregulation has been implicated in the development and progression of various malignancies, including prostate cancer [20]. Investigating AHR signaling in the context of ethnic disparities is crucial, as the interaction between AHR and these pathways may contribute to the variability in prostate cancer development among different ethnic groups [21].

There is growing interest in targeting AHR as a therapeutic strategy in prostate cancer. Preclinical studies have demonstrated that AHR antagonists and modulators can effectively inhibit the pro-tumorigenic effects of AHR signaling in cancer models, including prostate cancer [22]. Given the elevated AHR activity observed in AA men, targeting AHR could be a promising approach to mitigating the aggressive nature of prostate cancer in this population. Additionally, the convergence of AHR and AR signaling presents a dual target for therapeutic intervention. Drugs capable of simultaneously modulating both AHR and AR signaling pathways could offer a more effective treatment strategy for prostate cancer patients, particularly those with castration-resistant prostate cancer (CRPC), who have limited treatment options [23].

In conclusion, while socioeconomic and environmental factors undoubtedly contribute to the disparities observed in prostate cancer outcomes, there is mounting evidence that intrinsic biological differences, particularly in AHR signaling, play a critical role. Understanding the ethnic differences in AHR signaling and its interplay with other key pathways, such as AR signaling, is essential for unraveling the complex mechanisms underlying these disparities. This knowledge could pave the way for the development of targeted therapeutic strategies tailored to the unique genetic and biological characteristics of different populations, ultimately reducing the persistent disparities in prostate cancer mortality.

## Review

The exploration of ethnic disparities in prostate cancer outcomes has increasingly focused on the molecular mechanisms underlying the observed differences between AA and Caucasian populations. Central to this investigation are the AHR and AR signaling pathways, which play crucial roles in the progression and severity of prostate cancer. This review consolidates current research findings on these pathways and delves into how genetic, environmental, and molecular factors contribute to these disparities, particularly in the AA population.

AHR signaling in prostate cancer

The AHR is a ligand-activated transcription factor traditionally known for its role in detoxifying environmental carcinogens, such as PAHs and dioxins [1]. Upon ligand binding, AHR translocates to the nucleus and forms a complex with the ARNT, which binds to XREs in target genes [2]. This binding induces the expression of cytochrome P450 enzymes, such as CYP1A1, which are involved in the biotransformation and elimination of toxic substances [3].

Recent research has expanded the understanding of AHR beyond xenobiotic metabolism, showing its involvement in critical cancer-related cellular processes like cell proliferation, differentiation, apoptosis, and immune modulation [4-5]. In prostate cancer, AHR has been implicated in regulating AR signaling, a key driver of tumor growth and progression [6]. Elevated AHR expression has been observed in androgen-independent prostate cancer cells, linking AHR to the transition to CRPC, a highly aggressive and treatment-resistant form of the disease [7-8].

AHR’s role in prostate cancer is complex, particularly when considering its interaction with AR signaling. AR is activated by androgens, such as testosterone and dihydrotestosterone (DHT), which then translocate to the nucleus to bind androgen response elements (AREs) in the promoter regions of target genes, leading to the transcription of genes responsible for cell growth and survival [9]. The persistence of AR signaling in prostate cancer cells, even after androgen deprivation therapy (ADT), is a significant challenge in managing the disease [10].

Mechanistic Insight

AHR and AR pathways interact at multiple molecular levels. In addition to forming complexes in the cytoplasm, AHR modulates AR's nuclear translocation and transcriptional activity by binding to AR co-regulators. This synergistic relationship enhances AR-driven tumorigenesis and contributes to hormone therapy resistance. Furthermore, AHR’s role in modulating the immune response creates an immunosuppressive tumor environment, particularly through the regulation of T-cell differentiation and macrophage activation, further contributing to aggressive cancer phenotypes in AA patients.

Ethnic variability in AHR signaling

Ethnic differences in AHR signaling have been identified as one of the potential contributors to disparities in prostate cancer outcomes. Studies have consistently shown that AA men exhibit higher levels of AHR expression in prostate cancer tissues compared to Caucasian men, leading to more aggressive tumor phenotypes and higher mortality rates [13]. These differences are not solely genetic but are compounded by socioeconomic and environmental exposures, such as increased contact with PAHs and dioxins, which are known AHR ligands [14].

Environmental and Socioeconomic Context

AA men are disproportionately exposed to environmental carcinogens due to factors such as occupational hazards and living in highly industrialized areas with lower air quality. For instance, jobs that involve handling industrial chemicals or living in areas with higher air pollution contribute to greater AHR ligand exposure. This exposure, when combined with genetic polymorphisms in the AHR gene, exacerbates tumor progression and increases the risk of developing CRPC.

Genetic polymorphisms in AHR further modify its function. Certain AHR variants alter its ability to bind ligands or regulate downstream signaling pathways, contributing to differences in how AA men respond to environmental carcinogens and how their prostate cancer progresses [15-16]. This genetic-environmental interaction is a key factor driving the more aggressive nature of prostate cancer observed in AA, leading to poorer clinical outcomes [17].

In contrast, Caucasian populations generally exhibit lower levels of AHR expression, which correlates with slower disease progression and a better response to treatments like ADT. Understanding these population-based differences in receptor activity is crucial for tailoring targeted therapies that consider both genetic and environmental factors.

AR signaling and ethnic disparities

The AR signaling pathway plays an essential role in prostate cancer, and significant differences in AR activity have been documented between AA and Caucasian men. AA men tend to express higher levels of AR in prostate cancer tissues, which contributes to the more aggressive nature of the disease in this population [20]. Additionally, polymorphisms in the AR gene, specifically variations in CAG repeat lengths, significantly impact AR activity [21].

Shorter CAG repeats in the AR gene, which are more common in AA men, are associated with increased AR transactivation potential. This leads to enhanced AR signaling, promoting tumor growth and treatment resistance [22-23]. The combination of shorter CAG repeats and higher baseline AR activity contributes to the aggressive progression of prostate cancer in AAs, resulting in increased resistance to ADT [24].

Furthermore, the interaction between AHR and AR signaling creates a synergistic effect in AA patients, enhancing tumor aggressiveness. AHR upregulates AR target genes that are involved in tumor proliferation, further complicating treatment strategies [25]. This suggests that AA men may benefit less from AR-targeted therapies, like ADT, compared to Caucasian men, necessitating alternative treatment approaches.

Comparative analysis of AA and Caucasian populations

Caucasian men generally exhibit longer CAG repeats in the AR gene, leading to lower AR transactivation potential and a less aggressive cancer phenotype. These genetic differences, along with lower levels of AHR expression, suggest that Caucasian men respond better to ADT and experience slower disease progression. In contrast, AA men not only face genetic predispositions (e.g., shorter CAG repeats in AR and higher AHR expression) but also experience higher environmental exposure to carcinogens, which together lead to poorer outcomes. These disparities highlight the need for ethnically tailored therapeutic strategies that take into account both genetic makeup and environmental risks.

Therapeutic implications and future directions

Targeting both AHR and AR signaling is a promising strategy to address the ethnic disparities in prostate cancer outcomes. AHR antagonists have shown potential in preclinical models by inhibiting pro-tumorigenic effects in AA men with elevated AHR activity [26-27]. Additionally, combining AR inhibitors with AHR modulators may provide a more effective treatment for patients with CRPC, particularly those of AA descent [28-29].

Further research

There is a need for large-scale genomic studies involving diverse populations to fully understand how genetic variations and environmental exposures interact to influence prostate cancer outcomes. Understanding these complex interactions will enable the development of personalized therapeutic approaches tailored to the unique genetic and environmental profiles of different populations [30].

Discussion

Analyzing ethnic disparities in prostate cancer outcomes reveals a complex interplay of genetic, molecular, and environmental factors contributing to the differences observed between AA and Caucasian men. This discussion examines the roles of the AHR and AR signaling pathways, the impact of genetic variability, and the potential for targeted therapeutic strategies to address these disparities.

AHR Signaling and Ethnic Disparities

AHR has long been recognized for mediating the toxic effects of environmental carcinogens. However, its emerging role in cancer biology, particularly prostate cancer, underscores its potential contribution to ethnic disparities in cancer outcomes [1-2]. AA men show higher levels of AHR expression in prostate cancer tissues compared to Caucasian men, which may drive more aggressive tumor behavior and poorer clinical outcomes [3]. This overexpression may be linked to both genetic factors, such as AHR gene polymorphisms, and environmental exposures to carcinogens like PAHs and dioxins, which are more prevalent in socioeconomically disadvantaged communities [4-5].

The interaction between AHR and AR signaling pathways provides critical insight into how these molecular mechanisms may contribute to the aggressive nature of prostate cancer in AA men. AHR modulates AR signaling by affecting AR’s ability to translocate to the nucleus and bind to AREs, thereby influencing the transcription of genes involved in cell growth and survival [6]. This crosstalk between AHR and AR signaling may enhance the pro-tumorigenic effects of both pathways, leading to more rapid disease progression and resistance to standard therapies such as ADT [7].

AHR’s role in immune regulation adds another layer of complexity to its impact on prostate cancer outcomes. AHR influences the tumor microenvironment by modulating the activity of immune cells, including T cells and macrophages, which are essential for anti-tumor immunity [8]. Ethnic differences in immune response, potentially driven by variations in AHR activity, may contribute to the differential tumor microenvironments observed between AA and Caucasian men [9]. For instance, AA men may experience a more immunosuppressive tumor microenvironment, characterized by higher levels of regulatory T cells (Tregs) and myeloid-derived suppressor cells (MDSCs), which could facilitate tumor growth and metastasis [10].

Genetic Variability and AR Signaling

Genetic polymorphisms in the AR gene have long been associated with prostate cancer risk and progression. AA transactivation potential and higher AR activity [11]. These genetic differences may underlie the more aggressive prostate cancer phenotypes observed in this population, as elevated AR activity drives increased cell proliferation, survival, and resistance to apoptosis [12].

These genetic factors also influence therapy response. AA men often exhibit greater resistance to ADT, which targets AR signaling, due to higher baseline AR activity [13]. This resistance poses significant challenges for effectively managing prostate cancer in this group, as standard therapies may be less effective in reducing tumor burden and preventing disease progression [14]. Thus, alternative therapeutic strategies that target the underlying genetic and molecular mechanisms in this population are needed.

In addition to AR, other genetic factors contribute to ethnic disparities in prostate cancer. Variations in genes involved in steroid hormone metabolism, such as CYP17 and SRD5A2, which are more prevalent in AA men, may influence androgen levels and AR signaling [15]. These genetic variations can lead to higher circulating levels of testosterone and dihydrotestosterone (DHT), further enhancing AR activity and promoting tumor growth [16]. This genetic predisposition, combined with environmental factors increasing exposure to androgenic compounds, highlights the complexity of managing prostate cancer in AA men.

Environmental and Socioeconomic Factors

The interaction between genetic predisposition and environmental exposures significantly shapes the prostate cancer landscape. AA men are more likely to be exposed to environmental carcinogens due to socioeconomic factors, such as living in industrialized areas with higher pollution levels and working in occupations involving toxic substances [17]. These exposures, combined with genetic susceptibility, may increase the risk of developing aggressive prostate cancer and contribute to the observed disparities in disease outcomes [18].

Socioeconomic status also affects healthcare access, which is a critical factor in prostate cancer outcomes. AA men are less likely to receive early screening and diagnosis, often presenting with more advanced disease at diagnosis [19]. Disparities in access to high-quality care, including advanced treatment options and clinical trials, further contribute to the poorer outcomes observed in this population [20]. Addressing these socioeconomic disparities is crucial for improving prostate cancer outcomes and narrowing the mortality gap between AA and Caucasian men (Table 1).

**Table 1:** Comparative analysis of genetic and environmental factors influencing prostate cancer outcomes. AHR: Aryl Hydrocarbon Receptor; AR: Androgen Receptor; PAHs: Polycyclic Aromatic Hydrocarbons; CAG: Cytosine-Adenine-Guanine.

Factor	African American Men	Caucasian Men	Impact on Prostate Cancer Outcomes
AHR Expression	Higher levels in prostate cancer tissues.	Lower levels.	May drive more aggressive tumor behavior in African American men.
AR Gene Polymorphisms	Shorter CAG repeats leading to increased AR activity.	Longer CAG repeats, typically associated with lower AR activity.	Shorter CAG repeats in African American men contribute to higher AR signaling and potentially more aggressive cancer.
Environmental Exposures	Greater exposure to PAHs and dioxins due to socioeconomic factors.	Less exposure in general.	Increased exposure in African American men may amplify AHR activity, leading to more aggressive cancer.
Access to Healthcare	Limited access, often presenting with more advanced disease.	Better access, earlier diagnosis.	Disparities in access lead to later-stage diagnoses and poorer outcomes in African American men.

Therapeutic Implications and Future Directions in Prostate Cancer Treatment

A deeper understanding of the molecular mechanisms underlying ethnic disparities in prostate cancer has significant implications for developing targeted therapies. Antagonists and modulators of the AR show promise, particularly for AA men, who may exhibit increased AR activity due to genetic and environmental factors [21]. Therapeutic strategies that inhibit AR signaling could reduce its tumor-promoting effects and improve clinical outcomes for this group [22].

Moreover, combination therapies targeting both the AR and AHR signaling pathways offer a more effective treatment approach for patients with CRPC, especially those with elevated AR activity driven by hereditary factors [23]. These therapies could address the prevalent issue of resistance to ADT in AA men and provide a more personalized treatment plan (Table 2), taking into account the distinct genetic and molecular profiles of this population [19].

**Table 2:** Therapeutic strategies targeting AHR and AR signaling. AHR: Aryl Hydrocarbon Receptor; AR: Androgen Receptor; ADT: Androgen Deprivation Therapy.

Therapeutic Approach	Target Pathway	Potential Benefits	Population Focused
AHR Antagonists	AHR Signaling	Inhibit pro-tumorigenic effects of AHR, potentially reducing aggressiveness of prostate cancer.	African American men with high AHR activity.
AR Signaling Inhibitors (e.g., ADT)	AR Signaling	Reduce AR-driven tumor growth, though resistance may occur in high AR activity cases.	Patients with high AR activity, particularly African American men.
Combination Therapy (AHR and AR)	Both AHR and AR	Address both signaling pathways to combat aggressive prostate cancer forms and overcome resistance to ADT.	High-risk populations with genetic predispositions for high AHR and AR activity.

Further research is needed to fully elucidate the role of AR in prostate cancer and its interactions with other signaling pathways. It is crucial to include diverse ethnic groups in large-scale genomic studies to identify additional genetic variations that may contribute to ethnic disparities in prostate cancer outcomes [20]. Additionally, more investigation is required to understand how environmental exposures interact with genetic susceptibility to influence the risk and progression of the disease.

## Conclusions

The pronounced ethnic disparities in prostate cancer outcomes, particularly the higher incidence and mortality rates among AA men, underscore the urgent need to investigate the biological mechanisms behind these differences. This review highlights the critical roles of the AHR and AR signaling pathways in the aggressive nature of prostate cancer within AA populations. Elevated AHR activity, combined with genetic variations and environmental exposures, may amplify AR signaling, leading to more aggressive tumor behavior and resistance to conventional treatments such as ADT. Addressing these disparities requires a comprehensive approach that includes developing targeted therapies to specifically inhibit both AHR and AR pathways. Additionally, strategies to reduce environmental exposures and improve access to high-quality healthcare are essential. Ongoing research into the molecular and genetic factors influencing these pathways is crucial for developing personalized treatments that can bridge the mortality gap and enhance outcomes for all prostate cancer patients, regardless of ethnicity. By focusing on these key areas, significant strides can be made toward achieving equitable prostate cancer care.
